# Sorption of SARS-CoV-2 Virus Particles to the Surface of Microplastics Released during Washing Processes

**DOI:** 10.3390/ijerph19010281

**Published:** 2021-12-28

**Authors:** Noemi Belišová, Barbora Konečná, Nikoleta Bachratá, Jozef Ryba, Alena Potočárová, Michal Tamáš, Anh Le Phuong, Ondrej Púček, Juraj Kopáček, Tomáš Mackul’ak

**Affiliations:** 1 Department of Environmental Engineering, Faculty of Chemical and Food Technology, Slovak University of Technology in Bratislava, Radlinského 9, SK-812 37 Bratislava, Slovakia; nbachrata@gmail.com (N.B.); michal.tamas@stuba.sk (M.T.); ondropucek@gmail.com (O.P.); tmackulak@gmail.com (T.M.); 2 Institute of Molecular Biomedicine, Faculty of Medicine, Comenius University in Bratislava, Sasinkova 4, SK-811 08 Bratislava, Slovakia; basa.konecna@gmail.com (B.K.); alena.potocarova@imbm.sk (A.P.); 3 Department of Polymer Processing, Faculty of Chemical and Food Technology, Slovak University of Technology in Bratislava, Radlinského 9, SK-812 37 Bratislava, Slovakia; jozef.ryba@stuba.sk; 4 Department of Chemical Engineering, Faculty of Environmental Chemistry and Technology, Centria University of Applied Science, Talonpojankatu 2, 671 00 Kokkola, Finland; phuonganh1902.le@gmail.com; 5 Biomedical Research Center–SAV, Institute of Virology, Dúbravská Cesta 9, SK-835 05 Bratislava, Slovakia; virukopa@savba.sk

**Keywords:** microfibers, washing processes, wastewater, SARS-CoV-2, sorption of virus particles

## Abstract

The research aims at washing processes as possible sources of microplastics, specifical microfibers in wastewater, and the behavior of the virus particles SARS-CoV-2 in wastewater after the washing process as well as their ability to sorb to the surface of microfibers, released from washing processes. The conclusions of the research point to the ability of the virus to attach to possible solid impurities such as textile fibers (microfibers) occurring in the sewer and to the ability of wash water to influence their possible occurrence in the sewer. The highest efficiency (more than 99%) of removal virus particles was after washing process, using liquid washing powder, and washing soda. These findings may gradually contribute to a better understanding of the behavior of the virus particles in the sewer.

## 1. Introduction

The sudden outbreak of the infectious disease COVID-19 in 2019, caused by a coronavirus SARS-CoV-2, stimulated interest in targeting a total of coronaviruses to an even greater extent than before [1,2]. By the half of November 2021, there have been 253,163,330 confirmed cases of COVID-19, including 5,098,174 deaths, reported by the WHO. In Slovakia, there have been 563,445 confirmed cases of COVID-19 with 13,598 deaths, reported by the WHO [3].

Structurally, the viruses contain single-stranded RNA, ranging in size from 60 nm to 220 nm (coronaviruses from 80 nm to 220 nm) [4,5]. It has been found that many positive cases for COVID-19 have diarrhea [6,7], wherein the RNA virus was detected in the stool, thereby enter to the sewer [8,9]. As the given virus is identifiable on surfaces even after a certain time, during decontamination resp. washing surfaces or objects, viruses enter wastewater. SARS-CoV-2 is an enveloped virus, which envelope is essentially a portion of the cell membrane consisting of a phospholipid bilayer [10,11]. The virus can be effectively inactivated using alcohol preparations based on ethanol or isopropanol (at least 60% ethanol-and over 70% isopropanol), but also classical soap (dissolving lipids and eliminating the virus envelope) [12,13].

The hydrophobic interaction of the soap with the viral envelope is not sufficient to inactivate the virus. The research concluded that it is possible to use soap to inactivate the virus and inhibit its multiplication. The research around [14] also confirms the occurrence of the coronavirus SARS-CoV-2 in human wastewater. Work can also work in a similar way when it contains a lot of detergents, i.e., household water, can have some effect on the inactivation of coronaviruses in wastewater [15,16].

Microplastic fibers, also known as microfibers, are the most abundant microplastic forms found in the environment. The main sources of microfibers in the environment are caused by washing and drying processes, whether in households or industrial laundries [17,18,19]. The microfibers released during washing processes enter the sewer, and thus into the wastewater treatment plants [20]. Polyester is one of the most common microfibers released from the washing processes due to its predominance in the textile industry [18]. Degradation of MPs into smaller plastic particles may enhance the adsorption of contaminants on microplastics because more of MPs’ surface area is exposed, and their chemical reactivity increases. Environmental conditions such as weathering, sunlight, pH, long exposure times, and hydrophobicity of porous organic polymers (POPs) may significantly influence the kinetics of adsorption of contaminants to microplastics [21].. The highest risk group of pathogens occurring in wastewater and surface water are viruses, which, due to their microscopic size, good distribution, and low infectious dose, represent the main source of infectious diseases [22]. Hydrophobicity is one of the most critical factors governing the adsorption of molecules and objects, such as virions, on microplastics surfaces [23]. In our study it has been found, that released microfibers from washing processes influence virus behavior in wastewater. For this reason, in the first part, we focused on the washing process—a possible source of microfibers in wastewater and subsequently behavior of virus particles of SARS-CoV-2 in these types of wastewater. The study can be used as a supplement to a better understanding of behavior of virus particles of SARS-CoV-2 in sewage wastewater.

## 2. Materials and Methods

### 2.1. Washing Processes

A blanket of 100% polyester was used as research object. The washing process was realized in a laboratory model of a washing machine (Camry CR8052) in three washing programs—60-min; (a) without washing powder; (b) with liquid washing powder (capsule form Ariel); (c) with liquid washing powder (capsule form Ariel—Ingredients: (>30% Anionic surfactants, 5–15% soap, <5% nonionic surfactants, phosphonates, enzymes, optical brighteners, perfumes, Alpha-Isomethyl Ionone, Butylphenyl Methylpropional, Citronellol, Coumarin, Hexyl Cinnamal, Hydroxyisohexyl 3-Cyclohexene Carboxaldehyde, Linalool), and washing soda (Luxon-Sodium carbonate decahydrate; we used 12 g of washing soda for medium-hard water as recommended by the manufacturer). The temperature of the washing process was 40 °C and the volume of washing water was 15 L. The captured wastewater from the washing process was additionally filtered by vacuum filtration, with a filter size of 0.22 μm (MCE Membrane -Nitrocellulose membrane). The filters after vacuum filtration were dried at 40 °C in a dryer and subsequently considered. The difference between the weight of the filter before and after vacuum filtration we further stated when calculating the microfibers concentration (mg/L). The microfibers released during washing processes were used for experiment to determine the ability to sorb of SARS-CoV-2 virus particles to the surface of microplastics. The wastewater we used for analyzing chemical parameters.

### 2.2. Analyzes of Chemical Parameters in Water after Washing Process

Wastewater after washing process was analyzed using Hach sets; proceeded exactly according to the enclosed instructions. For analyzing phosphorus, ammonia, pH, conductivity was evaluated using a portable Hach SL1000 parallel analyzer, and cuvette tests for nitrate, calcium, magnesium, water hardness, and COD were evaluated using a DR6000 spectrophotometer.

### 2.3. Ability to Sorb SARS-CoV-2 Virus Particles

A total of 8 mg of microfibers were distributed to separate 50 mL falcon tubes. Furthermore, 2 mL of inactivated virus SARS-CoV-2 (strain Slovakia/SK-BMC5/2020, provided by Slovak Academy of Sciences, available at https://www.european-virus-archive.com/virus/sars-cov-2-strain-slovakiask-bmc52020 (accessed on 22 November 2021) was added to the 600 mL of tap water (typical room temperature) and 45 mL was dispensed to falcon tubes for each washing process. Similarly, the washing water was distributed to separate 50 mL falcon tubes and the same inactivated SARS-CoV-2 virus was added equally to each tube. Subsequently, the solutions were mixed by constant shaking for 2 h to simulate absorption. After mixing, the fibers were filtrated through standard chemical filtration paper and 4 g of PEG 8000 and 0.9 g of NaCl was added to each filtrate. After complete dissolution of PEG 8000, the mixture was centrifuged at 12,000× *g* for 99 min. Then centrifugated pellet was dissolved in 1 mL TRIzol and isolation of RNA according to the protocol of a manufacturer. The RNA pellet was finally resuspended in 60 uL of PCR-grade water. The presence of SARS-CoV-2 was assessed using the QuantiTect SYBR Green RT-PCR kit (Qiagen, Hilden, Germany) with specific primers for N1 and N3 gene as described by [24]. All experiments were performed in triplicate and the results presented are the average values obtained from the measurements.

## 3. Discussion

### 3.1. The Release of Microfibers during Washing Processes

The wastewater treatment plants (WWTPs) are in many studies defined as the source of releasing microplastics to the environment. In our study, synthetic materials were detected that were released during washing processes, and several procedures for washing processes were compared. Subsequent analysis of how many microfibers were released during household duties was carried. The variable parameter of washing processes was a washing agent or washing soda. The results are listed in Table 1 and show the average values of the individual washing cycles. The concentration of microfibers in the wash water was 1.617 g/L when we used only tap water without a washing agent. The highest concentration of microfibers was 2.2460 g/L in the wash water after using the liquid powder in capsule form together with washing soda. Microplastics, which may be present in the washing powder or washing soda, may also contribute to the higher concentration of MPs in the wash water [20].

### 3.2. The Efficiency of Washing Water to Remove SARS-CoV-2 Virus Particles

Microfibers released from washing processes to the wastewater were obtained and used for sorption tests with virus particles. First, inactivated SARS-CoV-2 virus particles were added to each type of wastewater after washing. Table 1 shows the effectiveness of the removal of virus particles from the wastewater. Values in Table 1 are average values for a given type of water. Tap water with the addition of virus particles is defined as “Start”. The lowest/zero virus particle removal efficiency occurred in the case of pure water without microfibers or washing powder after the washing process. It can be noticed that that such water does not affect the virus particles. Pure water without washing powder after the washing process with the addition of microfibers from washing process has a virus particle removal efficiency of 39%. This could be explained that the present microfibers have absorbed the virus particles and thus reduced their number in the water. The wash water after the washing cycle with the liquid detergent has a virus particle removal efficiency of 82%. The effectiveness was probably influenced by the composition of the liquid washing powder. The highest efficiency (reaching nearly 100%) of virus particle removal was the washing water with liquid powder and washing soda. This means that there was a very small amount of the virus particles in the water due to the combination of washing powder and washing soda, which ensured the nearly complete removal of virus particles.

Table 1 also shows the results of chemical parameters in washing water after washing cycles. The highest pH value was shown in the washing water after using liquid washing powder together with washing soda. The increase in pH, in this case, was caused by washing soda resp. sodium carbonate decahydrate, which is hydrolytically cleaved in an aqueous medium and raises the pH. The ammonia concentration was highest in the wash cycle with liquid washing powder and lowest in the wash cycle only with tap water. The composition of the washing powder contributed to the increase in the concentration of orthophosphates. Differences in calcium concentration in given types of wash water were low. The concentration of nitrate-nitrogen was highest when using liquid washing powder in the form of a capsule of 12.20 mg/L. Compared to the water samples from the other two cycles, this concentration was almost four times higher. The concentration was probably increased by the composition of the liquid washing powder. The last parameter monitored was COD. Washing water had the highest COD value after using liquid washing powder (4958 mg/L), followed by washing water after using liquid washing powder and washing soda (3742 mg/L) and washing water had the lowest COD after washing in clean water (204 mg/L). It follows that the greatest organic contamination was in the wash water after the use of the liquid washing powder. In this case, the cause of the organic contamination was the composition of the washing powder.

### 3.3. The Efficiency of Sorption of Virus Particles on the Surface of Microfibers

In the second part of this experiment, the ability of the virus particles to sorb onto the surface of microfibers, which are released during the washing process, was observed. In Table 2 we can see the efficiency of the expected sorption on a given type of microfibers. Values in Table 2 are average values for individual microfibers from a given washing cycle. The number of virus particles used for the experiment is in Table 2 are defined as “Start”. The microfibers (microfibers were removed from the dry polyester blanket before the first washing process) showed the lowest sorption efficiency (49%) before the washing process. The microfibers obtained after the washing cycle in water without washing reagent showed a sorption efficiency of the viral parts of 75%. Microfibers showed a slightly higher efficiency (77%) of sorption of virus particles after using liquid powder. The presence of the washing powder covering the fibers is likely to increase the sorption efficiency of the virus particles. In addition to the washing powder, impurities are also trapped on the microfibers, which increases the sorption surface, where they can trap virus particles. Microfibers from the washing process using liquid powder and washing soda have the highest efficiency (89%) of sorption of virus particles. The washing soda may be trapped on the surface of the microfibers and may contribute to the degradation of the RNA viral particles and at the same time RNA may be trapped onto the microfibers. From a comparable size of SARS-CoV-2 (diameter 60 (80)–140 nm) [25] and PET fibers (size length 350.93 to 2857.32 µm; width 2.67 to 16.04 µm it is left because the viral parts have enough space to capture. The size of microfibers was evaluated with the ImageJ software. Moreover, the lipophilic nature of the virus envelope plays in favor of the sorption of these particles onto PET microplastics, which are known as lipophilic contamination vectors [25,26].

### 3.4. The Sorption of SARS-CoV-2 on Microplastic Surface

In this time, we have limited quantity of information—how long is virus SARS-CoV-2 be able to active on the different surfaces [27]. In the sewerage system, the sorption of the present virus can take place directly on the surface of the microplast, for chemical or biological contamination/biofilm and for their combination too [21]. The microplastics or microfibers produced by the various washing processes can thus influence the concentration of viral particles in the sewerage system. The surface of microplastic and microfibers allows the adherence and colonization of microfibers, bacteria, and viruses also [28] because of their physic-cal porous structure, which can cause the transmittal pathogenic microorganisms [29]. According to the authors [30,31]. it is necessary to analyze in terms of monitoring SARS-CoV-2 not only in wastewater, but primary in sludge too, because virus particles can be able to sorb in the sewerage system to the surface of insoluble substances [32,33]. Particles of microplastics and microfibers present in wastewater. They may subsequently be part of primary sludge and thus participate on the sorb of virus particles that may subsequently affect the virus monitoring itself [32].

## 4. Conclusions

Our study, in the first part, was focused on some washing processes—as a source of microfibers in wastewater and the behavior of virus particles, SARS-CoV-2/SK-BMC5/2020 strain, in wastewater after the washing process. In addition, this study focused on the ability of microfibers to sorb virus particles on their surface. The highest concentration of released microfibers in the wastewater was after the wash cycle that used a liquid washing powder and washing soda (2470 g/L). In the second part of this study, the efficiency of influence of washing water on virus particles has been investigated. Washing water without powder was found to have the lowest removal efficiency (0%). The highest removal efficiency (>99%) was demonstrated by wastewater after washing using washing powder and washing soda. The microfibers from the washing cycle using liquid powder and washing soda showed the highest efficiency of sorption of virus particles on the surface of microplastics (89%). The results of this study can be helpful to understand the behavior of SARS-CoV-2 virus particles in wastewater. The methods of this study also can be helpful to increase the sensitivity of monitoring the occurrence of the SARS-CoV-2 virus.

## Figures and Tables

**Table 1:** The efficiency of washing water to remove SARS-CoV-2 virus particles and chemical parameters of washing water.

Washing Water	Concentration of Microfibers in Sample	Number of Copies of Virus Particles per Liter of Water	Sorption Efficiency	pH	NH_3_^+^	PO_4_^3−^	Ca^2+^	Mg^2+^	N-NO_3_	COD
	mg/L	Copy/L	%		mg/L	mg/L	mg/L	mg/L	mg/L	mg/L
Start	160	13,170,000	-							
Tap water without microfibers	-	-	0	7.33	0	0	71.8	17.2	12.76	0.74
Washing water with microfibers	160	8,015,000	39	7.90	0.41	1.30	78.70	17.50	2.07	204
Washing water with liquid powder	160	2,340,000	82	7.11	0.91	29	74.60	18.30	12.20	4958
Washing water with liquid powder and washing soda	160	0	>99	9.95	0.57	18.80	73.20	20.40	3.63	3742

**Table 2:** The efficiency of sorption of virus particles on the surface of microfibers.

Microfibers	Concentration of Microfibers in Washing Water after Washing Processes [g/L]	Number of Copies of Virus Particles per Liter of Water [Copy/L]	Sorption Efficiency [%]
Start		15,246,000	-
Before washing		7,763,000	49
Washing water	1.6170	3,794,000	75
Washing water with liquid powder	0.8910	3,433,000	77
Washing water with liquid powder and washing soda	2.2470	1,731,000	89
